# CNN-based object detection and growth estimation of plum fruit (*Prunus mume*) using RGB and depth imaging techniques

**DOI:** 10.1038/s41598-022-25260-9

**Published:** 2022-12-02

**Authors:** EungChan Kim, Suk-Ju Hong, Sang-Yeon Kim, Chang-Hyup Lee, Sungjay Kim, Hyuck-Joo Kim, Ghiseok Kim

**Affiliations:** 1 Department of Biosystems Engineering, Seoul National University, 1 Gwanak-Ro, Gwanak-Gu, Seoul, 08826 Republic of Korea; 2 Global Smart Farm Convergence Major, Seoul National University, 1 Gwanak-Ro, Gwanak-Gu, Seoul, 08826 Republic of Korea; 3 Research Institute of Agriculture and Life Sciences, Seoul National University, 1 Gwanak-Ro, Gwanak-Gu, Seoul, 08826 Republic of Korea; 4 Department of Convergent Biosystems Engineering, Sunchon National University, Suncheon, 540-742 Republic of Korea

**Keywords:** Imaging and sensing, Imaging

## Abstract

Modern people who value healthy eating habits have shown increasing interest in plum (*Prunus mume*) fruits, primarily owing to their nutritiousness and proven efficacy. As consumption increases, it becomes important to monitor work to prevent *Prunus mume* fruits from falling out. Moreover, determining the growth status of *Prunus mume* is also crucial and is attracting increasing attention. In this study, convolutional neural network (CNN)-based deep learning object detection was developed using RGBD images collected from *Prunus mume* farms. These RGBD images consider various environments, including the depth information of objects in the outdoor field. A faster region-based convolutional neural network (R-CNN), EfficientDet, Retinanet, and Single Shot Multibox Detector (SSD) were applied for detection, and the performance of all models was estimated by comparing their respective computing speeds and average precisions (APs). The test results show that the EfficientDet model is the most accurate, and SSD MobileNet is the fastest among the four models. In addition, the algorithm was developed to acquire the growth status of *P. mume* fruits by applying the coordinates and score values of bounding boxes to the depth map. Compared to the diameters of the artificial *Prunus mume* fruits used as the experimental group, the calculated diameters were very similar to those of the artificial objects. Collectively, the results demonstrate that the CNN-based deep learning *Prunus mume* detection and growth estimation method can be applied to real farmlands.

## Introduction

As of 2020, the global population was 7.8 billion, and it is expected to increase to 10 billion by 2050. In the meantime, the demand for agricultural products is also expected to increase rapidly. However, it is predicted that agricultural productivity will gradually decrease owing to the aging of the agricultural population. Moreover, rapid climate change and reducing agricultural land area have reduced productivity. Research into the incorporation of artificial intelligence and advanced sensor technology used in the 4th Industrial Revolution into the agricultural industry aims to overcome global problems in agriculture^1–4^. In the horticultural industry, cultivation and harvesting operations are still primarily dependent on manpower^2,5^, and the level of mechanization and automation technology utilized in the horticultural industry lags behind other industries^6^. Therefore, the need for state-of-the-art technology to collect, analyze, and manage growth during the growth process of horticultural crops is rapidly increasing. In particular, it is urgent to develop cutting-edge technology for growth analysis based on image information, and various studies are being conducted to address the situation^7–10^.

Since the 2000s, the popular perception of horticultural crops rapidly changed from simply fruits and salad vegetables to a functional key role in human health. Consequently, the cultivation and consumption of functional horticultural crops have rapidly increased^11^. Among numerous horticultural crops, *Prunus mume* fruit (plum) is highly sought after because of its rich nutrients, including minerals, vitamins, and organic acids. They are also known as alkaline fruits that relieve fatigue by promoting the metabolism of sugars^12,13^. Moreover, it has been reported that the *Prunus mume* fruit has antibacterial effects, it is regarded to be conducive in improving the constitution of modern people^12–17^. Adachi et al.^18^ reported that consistent ingestion of *Prunus mume* juice can suppress cancer in tumor-bearing hosts due to the anti-cancer components of *Prunus mume*, which verifies the health-promoting effects of *Prunus mume*. Go et al.^19^ investigated the relationship between the toxicity and metabolic changes of a chemical component called amygdalin in *Prunus mume* fruit. The effect of amygdalin metabolites on cytotoxicity was very small; however, it helped metabolism when consistently ingested. Additionally, Jung et al.^17^ conducted several clinical trials in rats to study the immunity-enhancing effect of fermented *Prunus mume* fruit. It was found that the fruit promotes probiotic formation in the bronchi of rats (*Bordetella bronchiseptica*), which also affects the immune function of human mRNA.

*Prunus mume* fruit is small, and its size during the harvest season is 3–4 cm^20^. Compared to other horticultural crops, a high yield of *Prunus mume* fruit can be harvested from a single tree^20^, so a considerable labor force is required during cultivation^21^. In addition, the damage caused by pests increases each year during the harvest season, and the technology to control the damage is still insufficient^22^. The primary cause of harmful pests of *Prunus mume* is known as peach seedlings (*Eurytoma Maslovskii*)^22,23^. Before the ovarian barrier of *Prunus mume* fruit hardens, eggs are spawned in the *Prunus mume* fruit, preventing the growth of the *Prunus mume* fruits^23^. Generally, adult insects spawn eggs in the *Prunus mume* fruit approximately two months before the harvest season. Therefore, it is critical to continuously monitor the growth of *Prunus mume* during this period when its flesh grows. However, since a single tree produces a very high yield and its size is relatively small, it is significantly difficult to continuously monitor the growth status manually through labor, such as counting the number of objects or analyzing the size of fruits.

Numerous studies are being actively conducted to analyze the growth of horticultural crops, and it involves automated image processing to replace existing manual work^24–27^. The monitoring system can perform various tasks automatically through the application of advanced technologies, such as object detection and size recognition of fruits. Recently, owing to the rapid development of artificial neural networks grafted into computing systems and imaging fields, deep learning-based studies have been actively conducted in various fields ^28–30^. In the agricultural industry, deep learning-based research is being applied rapidly, particularly to the research of agricultural robots and the growth estimation of crops^31–33^.

Compared to existing machine learning-based techniques in the imaging field, deep learning-based techniques exhibit significantly improved classification and detection performance^33–35^. Because CNNs are optimized for large amounts of image information, CNNs have been rapidly applied to image classification and detection, and have exhibited excellent performance^25,26,36^. In addition to the region-based convolutional neural network (R-CNN) method^37^, fast R-CNN^38^, faster R-CNN^39^, and mask R-CNN^37,40^ networks have also been developed and applied to various areas of study, such as disease and pest diagnosis, growth analysis, and fruiting analysis in the horticultural industry^41^. Specifically, Faster R-CNN^39^ is a two-stage method that extracts the feature map from the initial image through the convolution network, transfers it to the region proposal network (RPN), and resizes the box size to be fully connected using region of interest (ROI) pooling.

In addition to the R-CNN-based model that goes through two distinct steps, one-stage models such as single-shot multi-box detection (SSD)^42^ and Retinanet^43^ are being studied as the models can, simultaneously locate and classify the network. The SSD network was developed as a one-stage detection model to detect objects using a multi-scale feature map and a small kernel, with a fast detection speed compared to two-stage detection models. Retinanet introduced the concept of a feature pyramid network (FPN) and focal loss to enhance the learning contribution to difficult examples, thereby improving the performance. Subsequently, the EfficientDet^44^ model emerged, which is an object detection model that focuses on efficiency by minimizing the model size to maximize performance. The existing EfficientNet, which is used for classification, is used as a backbone network, and features are fused using a bidirectional feature pyramid network (Bi-FPN). The compound scaling technology proposed by EfficientNet refers to a method of increasing the model size and computational amount by considering various factors (input resolution, depth, width) at the same time. In addition, different input features have different resolutions, so a simple but more effective bi-directional FPN structure which takes different degrees of contribution to the output features is adopted, which broke out of the conventional FPN structure and resulting in huge improvement of detection performance. Different from the conventional FPN structure, Rasti et al.^27^ applied a deep learning-based object detection method, including transfer learning, to locate crops and estimate their growth stages. The constructed deep neural network is a convolutional neural network (CNN), and the weights finely adjusted using ImageNet were applied to the growth stage estimation. Moreover, Teimouri et al.^28^ presented a deep learning-based algorithm that automatically estimates the growth stage of eighteen different weed species. Accordingly, the application of a deep learning technique based on a convolutional neural network to estimate the initial growth status of eighteen weed species showed high accuracy of 78%, demonstrating that it can be applied to crops and weeds to estimate growth stages.

Nevertheless, in the case of *Prunus mume*, there is still insufficient research on growth stage measurement, including the application of pest control technology with advanced imaging technology. However, some studies have focused on the growth analysis of *Prunus mume.* Jang et al.^45^ presented a research result to recognize *Prunus mume* fruit and estimate its size by applying a three-dimensional (3D) imaging process. They experimentally predicted *the size of Prunus mume* fruit by using a size estimation program through a 3D imaging process to guide the timely control of *Eurytoma Maslovskii* that causes the most damage to *Prunus mume*. Moreover, Choi et al.^22^ conducted a study to reduce the average damage rate of *Prunus mume* caused by falling fruits caused by *Eurytoma Maslovskii* in Jeonnam, South Korea. Additionally, Lee et al.^23^ presented a cultivation environment monitoring system to determine the time to control *Prunus mume* to protect from pests. Accordingly, the authors measured the temperature, humidity, and illuminance of *Prunus mume* farms based on various sensors, and found that environmental conditions vary according to locations within the farm. Therefore, they developed a system that can continuously monitor *Prunus mume* efficiently by installing a data acquisition device.

In this study, we employed a deep learning-based RGBD object detection technology to measure and analyze the growth of *Prunus mume* fruit. To this end, an RGBD camera was used to collect photographs of *Prunus mume* fruits from outdoor farms. We performed image annotation and data augmentation, such as flipping images, image rescaling, image cropping, and adjusting brightness, to perform deep learning-based network training. Faster R-CNN Resnet 101, EfficientDet D4, Retinanet 101, and SSD MobileNet v.2 were used as networks for detecting *Prunus mume* objects, and transfer learning using the coco dataset was applied to all models. The size of the detected *Prunus mume* fruits was analyzed using depth information of the fruits from RGBD images. Lastly, a verification process was performed both indoors and outdoors to evaluate the deep-learning-based RGBD object detection technique and growth estimation method developed in this study. When the experiment was conducted indoors, artificial *Prunus mume* fruits and trees were used to verify their performance.

## Results

### Test results of prunus mume fruit detection

**Figure 1:**
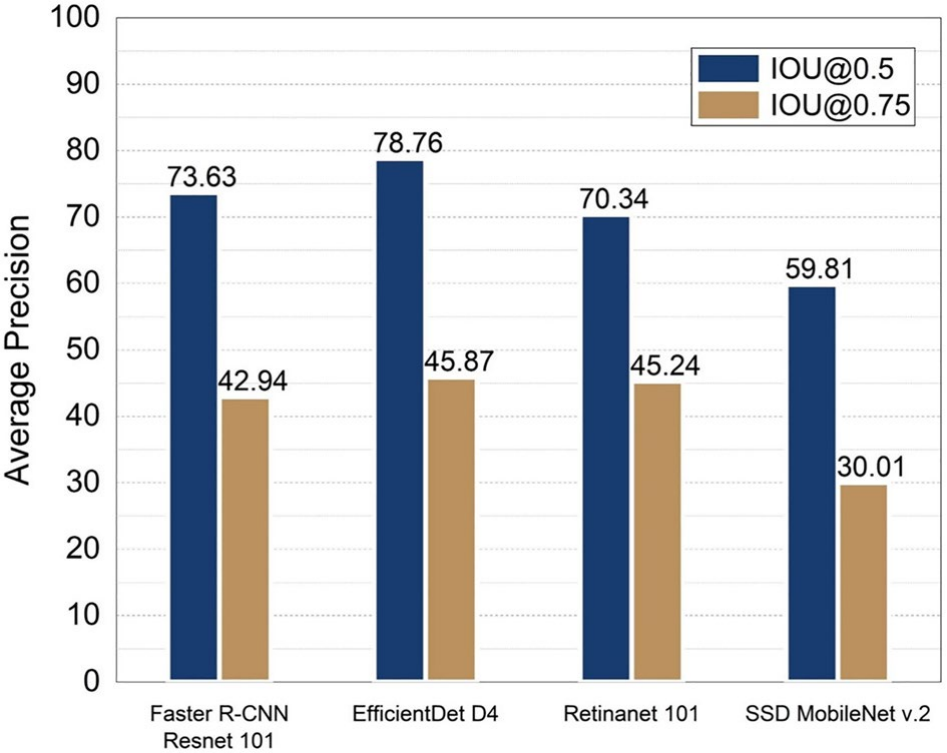
Performance comparison (AP) of models for each IOU.

Table 1 and Fig. 1 present the test results (average precision and inference time) of the four detection models used in this study. In the case of Faster R-CNN Resnet 101, the inference time was the slowest at 15.5 ms. However, the second-highest AP value (73.63 was obtained for the IOU threshold of 0.5. For the EfficientDet D4 model, despite its faster inference time than the Faster R-CNN Resnet 101 model, which had a value of 12.14 ms, the accuracy of the EfficientDet D4 model was the highest, with a value of 78.76 when the IOU threshold was 0.5. In particular, an AP value of 93.54 was obtained for large objects for the EfficientDet D4 model. In the case of the Retinanet 101 model, the inference time was 5.38 ms, which is approximately three times faster than Faster R-CNN Resnet 101. When the IOU threshold was 0.5 and 0.75, the AP value was 70.34 and 45.24, respectively. Despite being fast, Retinanet exhibited similar detection accuracy to Faster R-CNN Resnet 101. Lastly, in the case of the SSD Mobilenet v.2 model, the inference time was fastest among four models showing 2.96 ms per image, while its AP value exhibited the lowest detection performance, specifically, 59.81 when the IOU threshold was 0.5 and 30.01 when the IOU threshold was 0.75. The relatively light structure and small input size of the SSD MobileNet v.2 model improved the computing speed. However, it seems to be unsuitable for the *Prunus mume* fruit detection task compared to the rest of the models applied in this study.

**Table 1:** Test results of detection models.

Model	Input size	Inference time (ms /image)	AP				
			IOU:0.5	IOU:0.75	Small	Medium	Large
Faster R-CNN Resnet 101	1024	15.50	73.63	42.94	42.74	77.37	87.74
EfficientDet D4	1024	12.14	78.76	45.87	37.90	85.30	93.54
Retinanet 101	640	5.38	70.34	45.24	30.43	77.44	89.98
SSD MobileNet v.2	640	2.96	59.81	30.01	19.15	63.65	85.34

**Figure 2:**
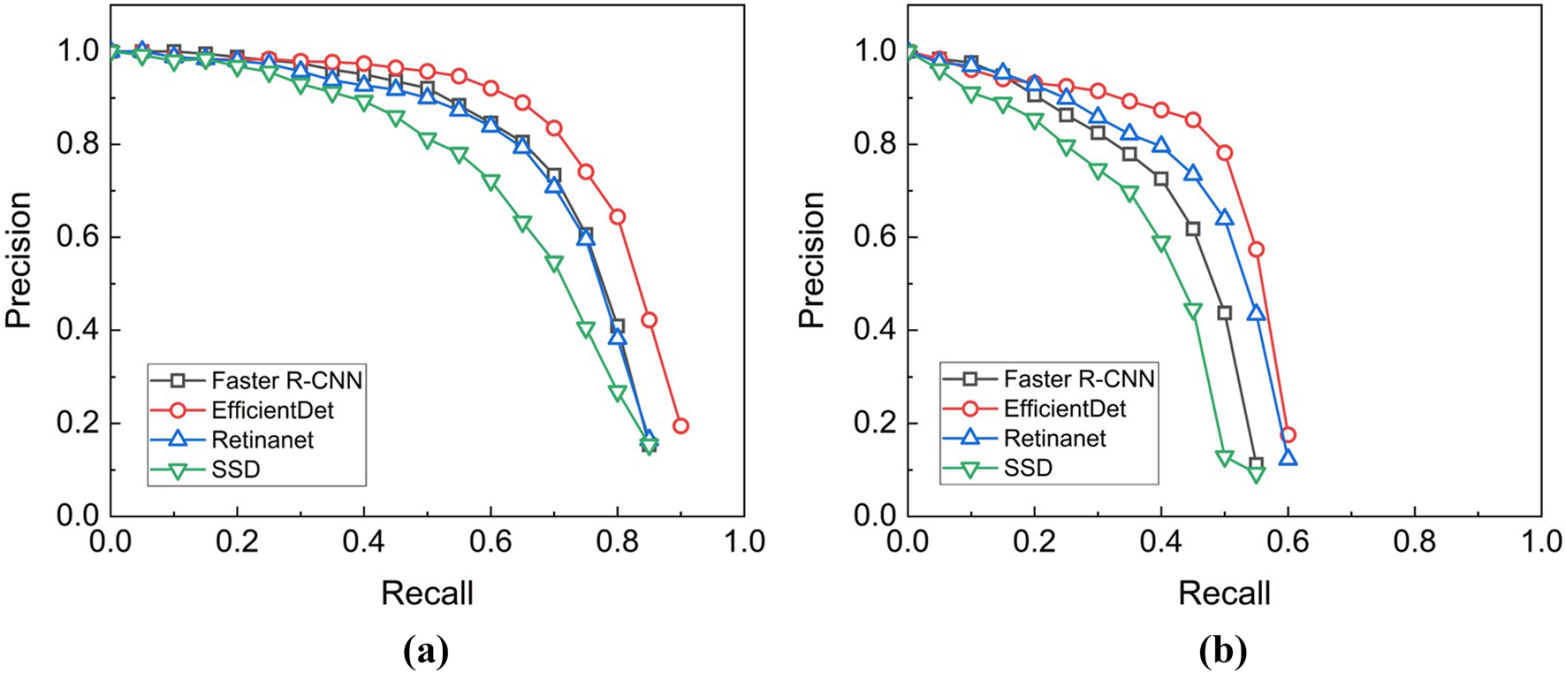
Precision-recall (PR) curve of models: (**a**) IOU: 0.5; (**b**) IOU: 0.75.

**Figure 3:**
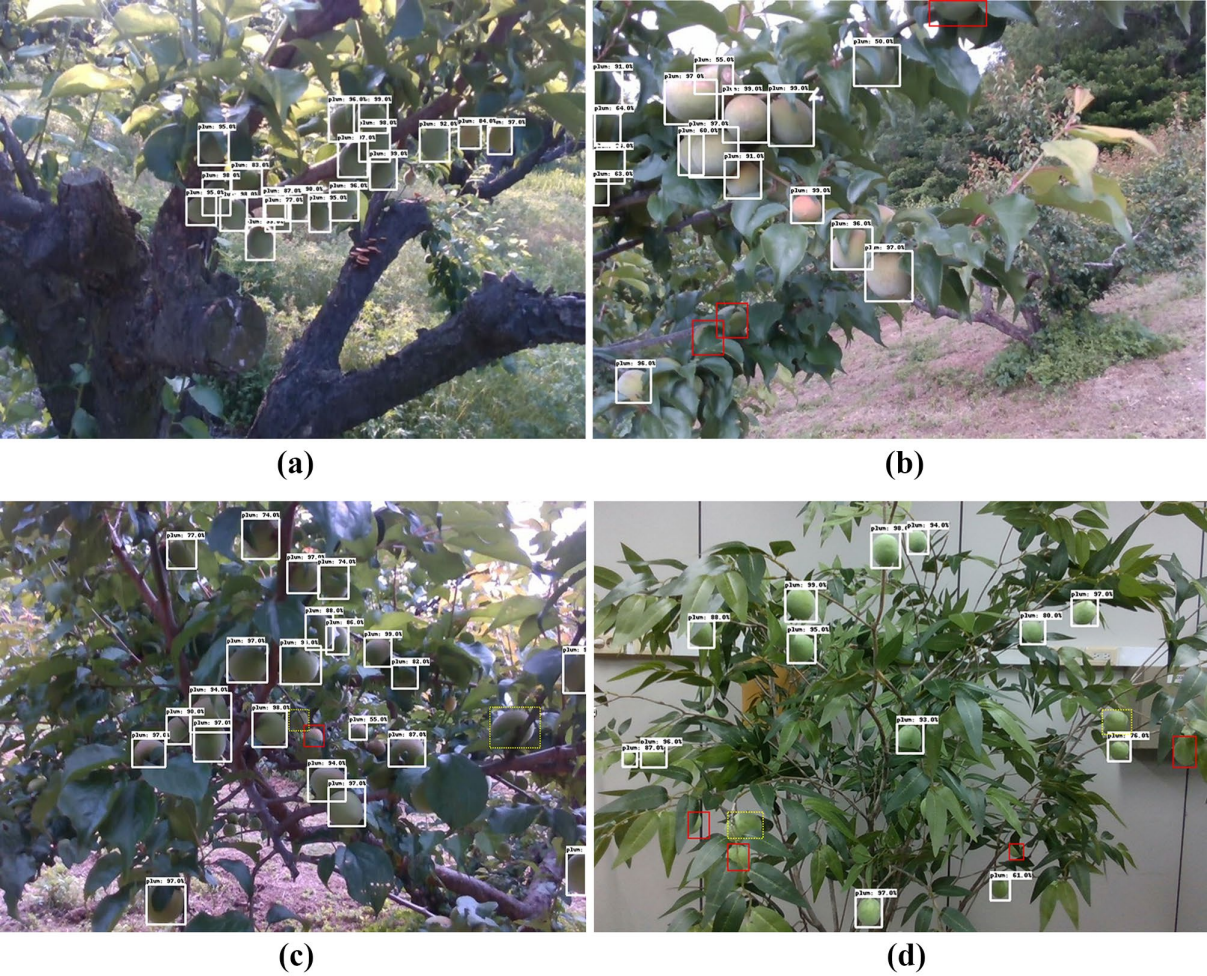
Detection results of EfficientDet D4 detection model.

Figure 2 illustrates the precision-recall (PR) curves of the detection models. In the case of Fig. 2a, the EfficientDet D4 model maintained a high precision, close to the value of 0.9, even in the high recall area when the IOU threshold was 0.5. However, in the case of other models, it can be seen that the precision decreases significantly when recall increases, such as Faster R-CNN, Retinanet, and SSD MobileNet., In particular, the SSD MobileNet v.2 model exhibited significantly lower performance compared to the other three networks. Figure 2b illustrates the PR curves of the four models when the IOU is 0.75. It is evident that its accuracy was significantly reduced compared to the accuracy when the IOU was 0.5. Figure 3 illustrates the detected results of *the Prunus mume* fruits using the EfficientDet D4 detection model developed in this study. The white solid box area in Fig. 3 represents normally detected objects (True Positive) that satisfy a score of 0.75 or higher. Evidently, all *Prunus mume* fruits that needed to be detected were successfully detected, as shown in Fig. 3a. As indicated in Fig. 3b, the objects boxed by the red line (True Negative) were not detected, which are undetected objects that do not need to be detected. The reason for this is that the annotation was not performed for those whose occlusion parts exceeded 50% or those that were too blurred to recognize in the initial annotation process, so it can be considered a normal detection result. However, when observing the part with the yellow dotted line in Fig. 3c,d, it can be seen that the object is not detected even though there is almost no occluded part. The yellow dotted line box indicates objects that should be detected but were not detected (false negative).

As shown in Fig. 3, despite its small object size compared to the entire image size, in most cases, it is clear that the *Prunus mume* fruits were generally well detected, except for these few undetected parts. Nevertheless, it still seems difficult to detect all of the objects, particularly in the case of those whose parts were occluded more than half due to surrounding bushes, which were marked with red boxes in Fig. 3, it is evident that the model could not detect. It is assumed that objects are undetected because of their small size compared to the entire image size. As indicated in Table 1, the AP values of the small objects, which were defined smaller than 22 × 22 pixels, were significantly lower than those of other pixel sizes. We assume that this is also due to reasons such as erroneous detection of the leaves of trees as *Prunus mume* fruits and many obstructions mentioned above. Therefore, an image cropping method was applied to improve the detection performance of small objects. Original images of 1280 × 720 pixels were cropped into 960 × 540, 640 × 360, and 320 × 180 pixels, and the models were trained and evaluated for each condition.

**Figure 4:**
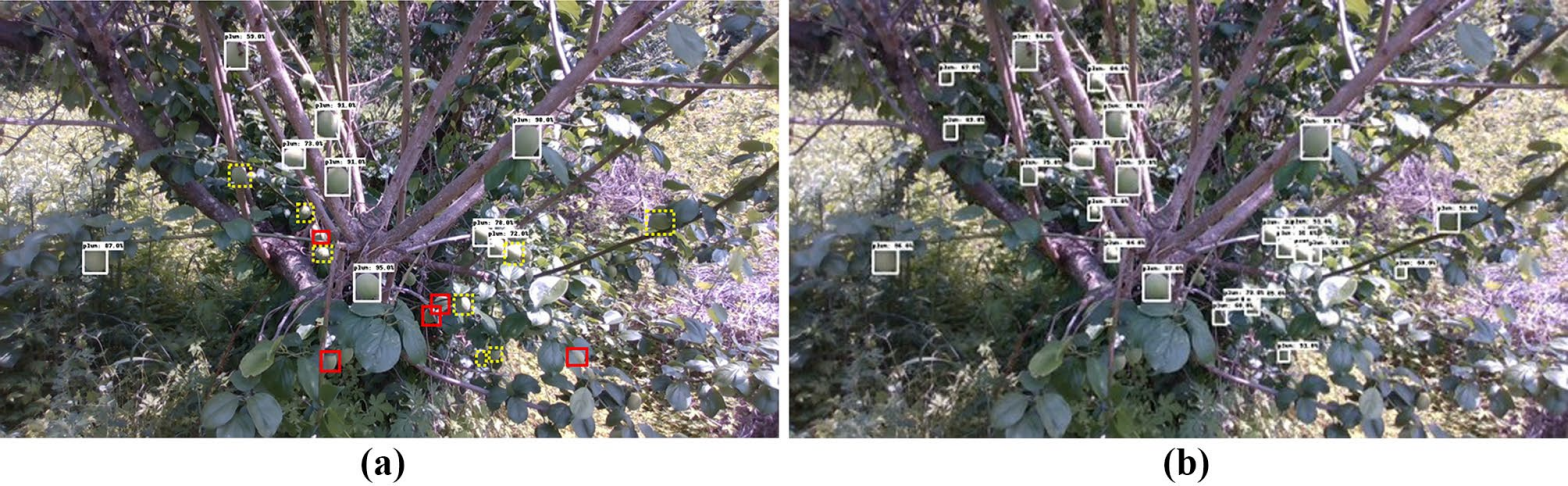
Detection results of same *prunus mume* image: (**a**) Before applying cropping method; (**b**) After applying cropping method (640 $\times$ 360).

Table 2 lists the results of the EfficientDet D4 model for the four different cropping conditions. The model based on the 960 × 540 pixel image exhibited higher AP values than the model without cropping in all AP cases. In particular, the AP value of small objects, which was 54.28, increased by 16 compared to the existing EfficientDet D4 model, which had a value of 37.90, as indicated in Table 2. The 640 × 360 pixel image-based model did not show any significant difference in AP values, excluding the small object compared to the 960 × 540 pixel image-based model. However, the AP for small objects increased by approximately 6. In this manner, when cropping an image, the overall performance improved, particularly for small objects. Consequently, compared to the detection result of which cropping method was not applied, as seen in Fig. 4a, it is evident that more objects as well as yellow dotted boxed objects were detected after applying the cropped model, as seen in Fig. 4b. However, when scanning the original image using this cropping method, simply cropping without overlap may result in detection errors in objects across the end of the cropped window. Therefore, overlap between cropping windows according to the size of the objects is required when applying the cropping model to the original image. The application of such an overlap can increase the number of windows to be scanned, leading to an increase in processing time; therefore, it is necessary to select an appropriate method based on the speed and accuracy requirements of the task. Therefore, by considering the processing time and AP efficiency, the 640 × 360 pixel image-based model was considered as the most appropriate model for *the Prunus mume* fruit detection model, as well as for verifying the growth estimation process in this study.

**Table 2:** Average precision results of image cropping models.

Model (Cropped size)	Inference time (ms/image)	Input size	AP				
			IOU:0.5	IOU:0.75	Small	Medium	Large
EfficientDet D4 (1280 × 720)	12.1	1024	78.76	45.87	37.90	85.30	93.54
EfficientDet D4 (960 × 540)	51.2	1024	84.21	58.46	54.28	90.89	94.55
EfficientDet D4 (640 × 360)	109.6	1024	85.47	60.49	60.56	90.44	94.54
EfficientDet D4 (320 × 180)	291.3	1024	85.36	61.69	65.71	89.81	94.66

### Verification process for evaluating growth stages

**Figure 5:**
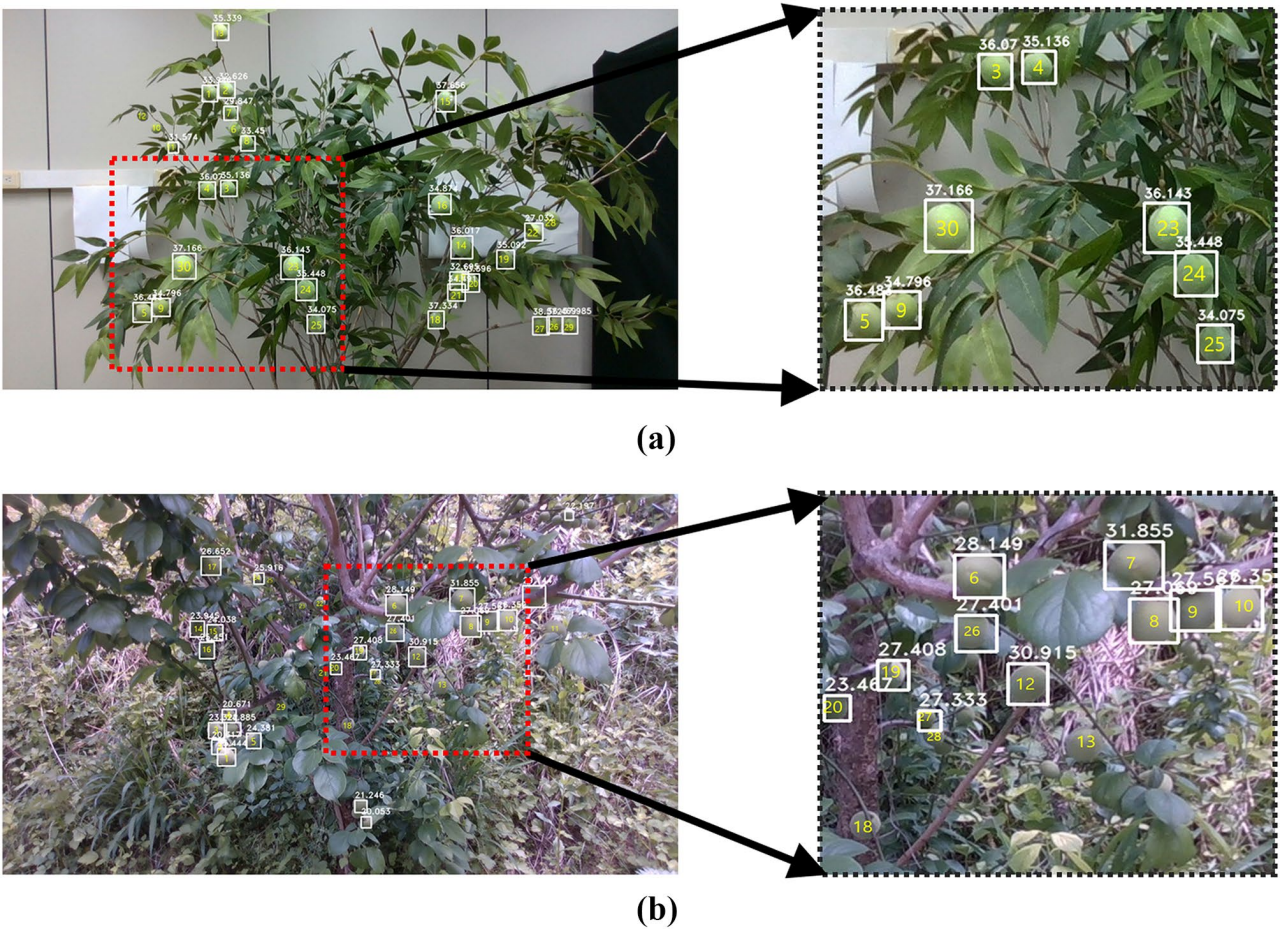
Calculation of each numbered objects’ diameter using depth information: (**a**) indoor; (**b**) outdoor test image.

In this study, we developed a method for evaluating *Prunus mume* fruit growth stages using the information of bounding box coordinates, which are the result of object detection, and depth values of RGBD images. Figure 5 illustrates the results of the growth evaluation by applying deep learning and 3D image processing techniques based on RGBD images. Moreover, Fig. 5a,b show the results of the growth estimation for each numbered target, indoor artificial fruits, and real *Prunus mume* fruits grown outdoors, respectively. For the detection model, EfficientDet D4 cropped with 640 × 360 pixels, which was confirmed to have the highest detection precision (AP) when detecting *prunus mume* fruits was adopted. During the application of the evaluation technique for growth information, objects that were not detected (false negative) and those that were not subject to detection because more than half of their images were lost due to bushes or other objects (True Negative) were excluded from the evaluation target. The method was applied only for the *Prunus mume* fruits normally detected (true positive) with a score of 0.75 or higher. The growth information (diameter or area) calculated using the developed algorithm was compared with the actual measured size by Vernier calipers to verify the accuracy of the growth analysis technique. In Fig. 5, the white solid line represents the detected *Prunus mume* fruits, and white numbers above each box indicate the length (long axis) of the objects in millimeters ($mm$).

Table 3 lists the actual size, estimated size, error value, and error rate classified into small, medium, and large objects depending on the pixel sizes in the images for estimating the sizes of artificial fruits shown in Fig. 5a. The ranges of the object’s pixel values were classified as follows: small objects as 0 × 0 to 22 × 22, medium objects as 22 × 22 to 34 × 34, and large objects as 34 × 34 or more. As listed in Table 3, only one out of four *Prunus mume* fruits was detected in a small area. Although the detection precision for small objects greatly increased following the application of the cropping method by 640 × 360 pixels, it was determined to have lower detection accuracy because one more step of regulation for detection was applied, using only the detection boxes whose score was 0.75 or higher. It can be seen that the errors and error rates between the actual measurement and predicted value of one small-sized object (Number 11) showing larger values occurring in medium and large sized objects, specifically, 2.07 mm and 6.15%, respectively. The average error and error rate of medium-sized artificial objects were 2.05 mm and 5.90%, respectively, which were lower than those of small-sized objects. In the case of large-sized objects, the average error and error rates had the lowest values of all sizes, which were 1.97 mm and 5.66%, respectively.

**Table 3:** Results of applying growth evaluation algorithm to an indoor image.

Size (pixel)	Object number	Measured diameter (mm)	Predicted diameter (mm)	Error (mm)	Error rate (%)
Small (0–22 × 22)	11	33.64	31.57	2.07	6.15
	Average	33.64	31.57	2.07	6.15
Medium (22 × 22–34 × 34)	1	35.26	33.95	1.31	3.72
	2	36.73	32.63	4.10	11.16
	3	34.23	35.14	0.91	2.66
	4	34.82	36.07	1.25	3.59
	5	36.03	36.48	0.45	1.25
	7	33.94	29.85	4.09	12.05
	8	35.03	33.45	1.58	4.51
	9	34.77	34.80	0.03	0.09
	13	34.73	35.34	0.61	1.76
	17	35.78	32.69	3.09	8.64
	18	34.02	37.33	3.31	9.73
	19	34.97	35.09	0.12	0.34
	20	34.96	33.60	1.36	3.89
	21	35.47	34.49	0.98	2.76
	22	34.55	27.03	7.52	21.77
	25	33.66	34.08	0.42	1.25
	26	34.83	36.43	1.60	4.59
	27	35.58	38.50	2.92	8.21
	29	34.52	37.99	3.47	10.05
	Average	34.94	34.47	2.05	5.90
Large(34 × 34 ~)	14	33.35	36.02	2.67	8.01
	15	35.66	37.66	2.00	5.61
	16	35.47	34.87	0.60	1.69
	23	34.60	36.14	1.54	4.45
	24	37.32	35.45	1.87	5.01
	30	34.05	37.17	3.12	9.16
	Average	35.08	36.22	1.97	5.66

For the *Prunus mume* fruits in the outdoor farm shown in Fig. 5b, the results of applying the technique for evaluating growth information were analyzed, as listed in Table 4. The actual measurement and predicted values of 20 objects, excluding nine undetected objects, were compared, and their errors and error rates were analyzed. Out of the 20 detected objects, five, ten, and five objects were identified to be small, medium, and large, respectively. Seven out of nine undetected objects were small, and the other two were medium-sized. In Fig. 5b, it was confirmed that the average diameter of fruits was 27.60 mm, which was approximately 8 mm smaller than the average size of *Prunus mume* fruits during the harvest season. Moreover, fruits had a variety of sizes, ranging from 18.16 mm to 31.62 mm in actual diameters. As indicated in Table 4, the average value of actual measurements and predicted values of the fruits having the small-sized areas were 28.06 mm and 24.96 mm, respectively, exhibiting an error of 3.10 mm. The error rate of small-sized fruits was 11.26%, which had the highest percentage for all three sizes. The actual measurements and predicted values of the fruits included in medium-sized area were 26.80 mm and 25.28 mm, respectively, and the average error and error rate were analyzed to be 2.01 mm and 7.44%, respectively. In the case of large-sized objects, the actually measured average value was 29.74 mm and the predicted value was 28.20 mm. Their average error and error rate were 1.92 mm and 6.27%, respectively. Resultantly, the large-sized fruits were confirmed to have the lowest error value of the three divided pixel-based sizes.

**Table 4:** Results of applying growth evaluation algorithm to an outdoor image.

Size (pixel)	Object number	Measured diameter (mm)	Predicted diameter (mm)	Error (mm)	Error rate (%)
Small (0–22 × 22)	4	25.17	20.67	4.50	17.88
	19	29.76	27.41	2.35	7.90
	20	27.31	23.47	3.84	14.06
	24	27.64	25.92	1.72	6.22
	27	30.44	27.33	3.11	10.22
	Average	28.06	24.96	3.10	11.26
Medium (22 × 22–34 × 34)	1	25.58	26.44	0.86	3.36
	2	22.40	20.32	2.08	9.29
	3	27.55	23.30	4.25	15.43
	5	28.02	24.38	3.64	13.00
	12	29.28	30.91	1.63	5.57
	14	24.20	23.95	0.25	1.03
	15	27.00	24.04	2.96	10.96
	16	27.27	25.45	1.82	6.67
	17	29.07	26.65	2.42	8.32
	26	27.61	27.40	0.21	0.76
	Average	26.80	25.28	2.01	7.44
Large (34 × 34–)	6	31.40	28.15	3.25	10.35
	7	31.62	31.86	0.24	0.76
	8	28.28	27.07	1.21	4.28
	9	31.75	27.57	4.18	13.17
	10	25.64	26.35	0.71	2.77
	Average	29.74	28.20	1.92	6.27

Regarding all the images used during the process of verifying the method for evaluating growth stages, it was found that the detection rate of small-sized objects was 67.19%, which exhibited the lowest value. Meanwhile, the detection rates of medium-sized and large-sized objects were 81.58% and 90.91%, respectively. It was also shown that the diameter error rate of the small objects was 8.05%, and those of the medium and large objects were 7.26% and 6.82%, respectively, indicating that the diameter error rate decreases when the size of objects increases. In summary, when the sizes of objects compared to the overall image size decrease, the detection rates decrease, which inevitably reduces the scores of the detected boxes and increases the diameter error rates.

**Figure 6:**
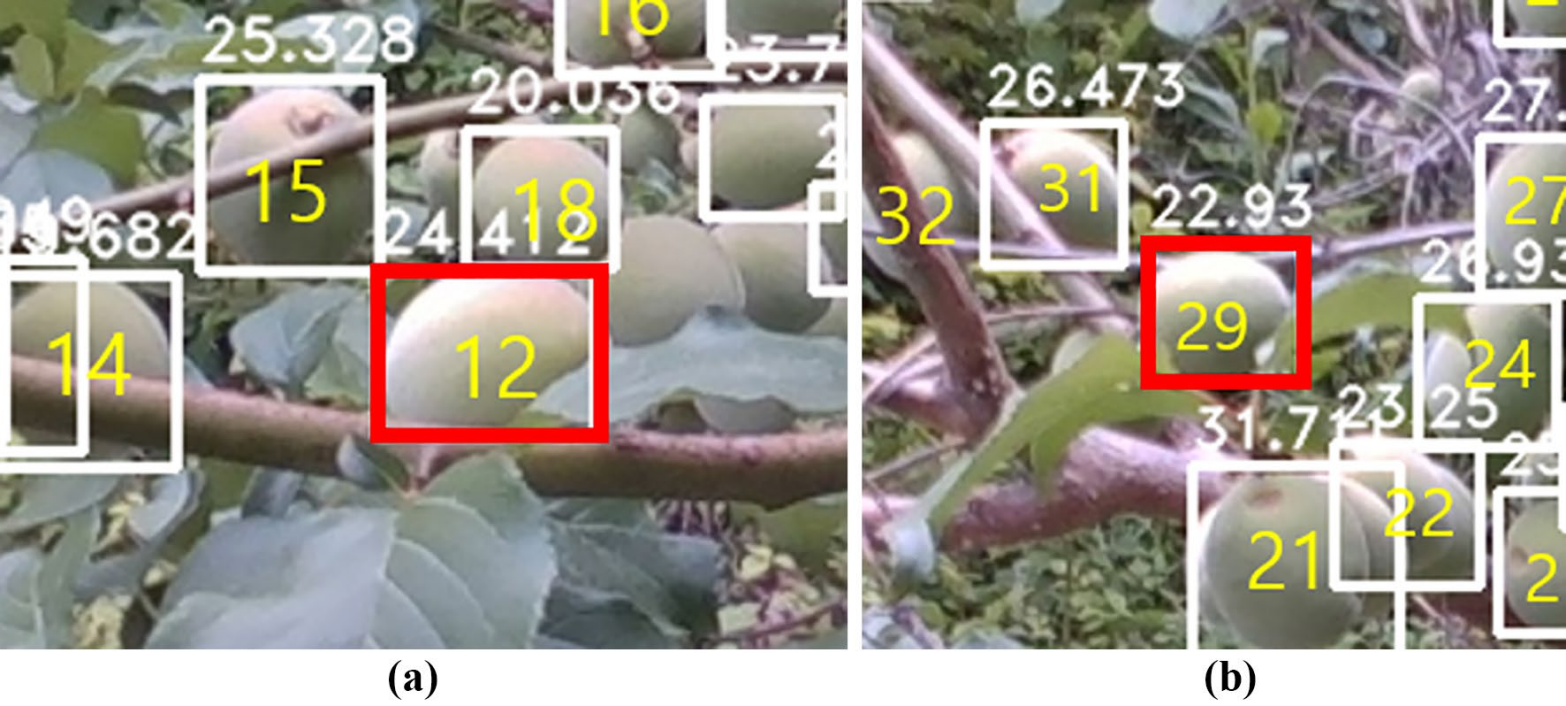
Case of losing growth information of some *prunus mume* fruits (Number 12 and 29) due to adjacent fruits or surrounding bushes.

However, exceptionally analyzed values of some objects with abnormally high errors and error rates were confirmed in the verification process. As listed in Table 3, the average error rate of diameter in the medium-sized area was 5.90%; however, in the case of *Prunus mume* fruits numbered 7 and 22, the average error rates were 12.05% and 21.77%, respectively, exhibiting a significant difference from the average value. Similarly, in Table 4, it can be confirmed that the error rates of some *Prunus mume* fruits were 15% or higher, which is significantly larger than the average error rate. For instance, in the case of *Prunus mume* fruits numbered 12 and 29 shown in Fig. 6 drawn with a red solid line box, it was normally well detected because less than 50% was lost due to adjacent objects or surrounding bushes (True Positive). However, it can be seen that the occlusion part was not included in the white solid box area. It was determined that the growth information of the occluded part was not included in the growth information analysis. Since the growth information of the occluded part was not included, large errors occurred for some objects during the application of the method.

## Discussion

In this study, deep learning-based object detection techniques were proposed for detecting *Prunus mume* fruits based on RGBD images. The following techniques were applied: Fast R-CNN, EfficientDet, Retinanet, and SSD MobileNet. These deep learning-based detection models were used as training datasets with images collected from actual *Prunus mume* farms, and the training process was conducted by dividing a total of 719 images and 12,773 *Prunus mume* fruits. Considering that the colors of the fruits are similar to those of the surrounding bushes or backgrounds and that the size is very small compared to the entire image size, a technique for cropping images was applied to improve detection precision (AP), which significantly improved the detection precision of objects for small-sized objects. As a result of the detection part, the average precision values (AP) of detection accuracy ranged from 70.34% to 85.47%, whereas in the case of the EfficientDet D4 model, which exhibited the highest AP value among the four detection models, the detection rate was 78.76. As such, it was determined that in the case of objects such as *prunus mume* fruits, the EfficientDet had highest AP value due to its compounding scaling characteristics and adopted bi-FPN structure unlike other models. Although the EfficientDet D4 model identified in our study may be useful detection model for *prunus mume* fruits, it is expected that it may show different detection performances for other fruits. Therefore, when developing a detection model for specific fruit, it is considered to develop a unique detection model appropriately.

In addition, the AP values of small, medium, and large objects in the case of EfficientDet D4 model were found to be 42.74, 85.30, and 93.54, respectively. In addition, by applying the image cropping technique to the EfficientDet D4 model, it was confirmed that when the IoU threshold was 0.5, the AP value increased to 84.21 compared to 78.76 before the image cropping technique was applied. Moreover, the AP values of small, medium, and large objects also improved to 60.56, 90.89, and 94.55, respectively. Applying the image cropping technique seems to be unsuitable for some object detection models because the inference time increases by approximately nine times. However, as we intended to analyze the growth information precisely based on the accurate (x, y) coordinates of the bounding boxes with scores higher than 0.75, the proposed object detection model was determined to be suitable for application to analyze the growth information according to the growth stages of real *Prunus mume* fruits.

The EfficientDet D4 (640 × 360) model, which had the highest detection accuracy (AP) value, was used to verify the technique for evaluating growth developed in this study, and the predicted diameters were compared to the actual diameters of the *P. mume* fruits to verify its accuracy. For all *prunus mume* fruits used in the verification process, the detection rate of small-sized objects was the lowest, with a value of 67.19%, and the detection rates of medium and large-sized objects were 81.58% and 90.91%, respectively. The diameter error rate of small-sized objects was the highest, with a value of 8.05%, while those of medium-and large-sized objects were 7.26% and 6.82%, respectively. It was determined that when the size of the object compared to the overall image size decreased, the detection rate also decreased. This low detection accuracy value also reduced the score of the bounding box, causing the error rates to increase.

Lastly, we estimated the overall quality of the RGBD sensor used in the experiment, which indicated that the results were sufficient for application in actual farms. Owing to some hardware limitations and the difficulties in detecting *Prunus mume* fruits with colors similar to the surrounding background, the deep learning-based object detection model proposed in this study should be continuously supplemented, and model optimization should be continually studied and developed for specific applications by carefully considering the speed-accuracy trade-off. Furthermore, it is necessary to perform studies in the future to improve speed and accuracy by modifying various hyper-parameters to suit each *Prunus mume* image to enable more accurate detection in the vegetation environment.

## Methods

### Image collection and pre-processing

**Figure 7:**
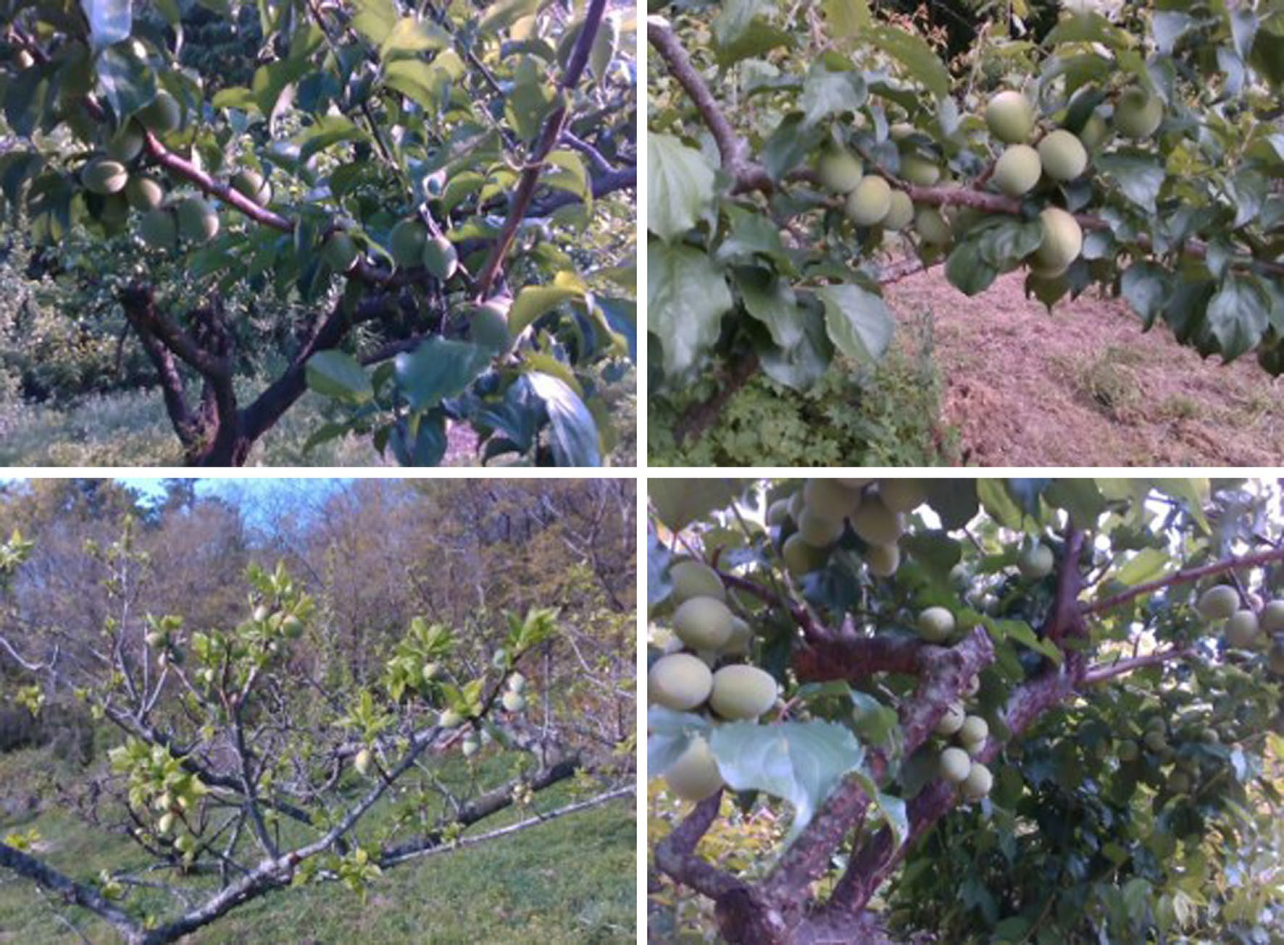
*Prunus mume* images acquired by the RGBD sensor.

Figure 7 illustrates green *Prunus mume* fruits during the harvest season. In this study, we attempted to evaluate the growth of these fruits using a CNN based 3D image processing technique. First, we collected 800 photographs of *Prunus mume* images from two local farms (Suncheon City, Republic of Korea) every two weeks from April to May 2020. We acquired the *prunus mume* fruits and trees’ images in a legitimate way by contacting farm owners in advance, attaining permission to acquire *prunus mume* photographs. Also, the experimental research and field studies on *prunus mume* plants in our study were complied with relevant institutional, national, and international guidelines and legislation. The photos were captured using an RGBD camera (D435i, Intel RealSense Inc., USA), which comprised of RGB and depth sensors. The pixel resolutions and field-of-views of RGB and depth sensor were 1920 × 1080, 1280 × 720, and 69.4° × 42.5° and 86° × 57°, respectively. The *Prunus mume* photos were captured during the daytime from 11 am to 4 pm throughout the entire measurement period to acquire photos under different lighting conditions. The image captured by the camera includes both RGB and depth information simultaneously, meaning that when acquiring RGB and depth images, the photo must contain the same information at the same pixel coordinates. Matching RGB and depth images is important for extracting accurate growth stages of detected *Prunus mume* fruits. Therefore, the depth image’s field-of-view should be adjusted precisely to acquire the morphological information of fruits correctly using their depth information. Python (ver. 3.7, Corporation for National Research Initiatives, England, Europe) was used as the programming language for this calibration process, including field-of-view correction.

**Figure 8:**
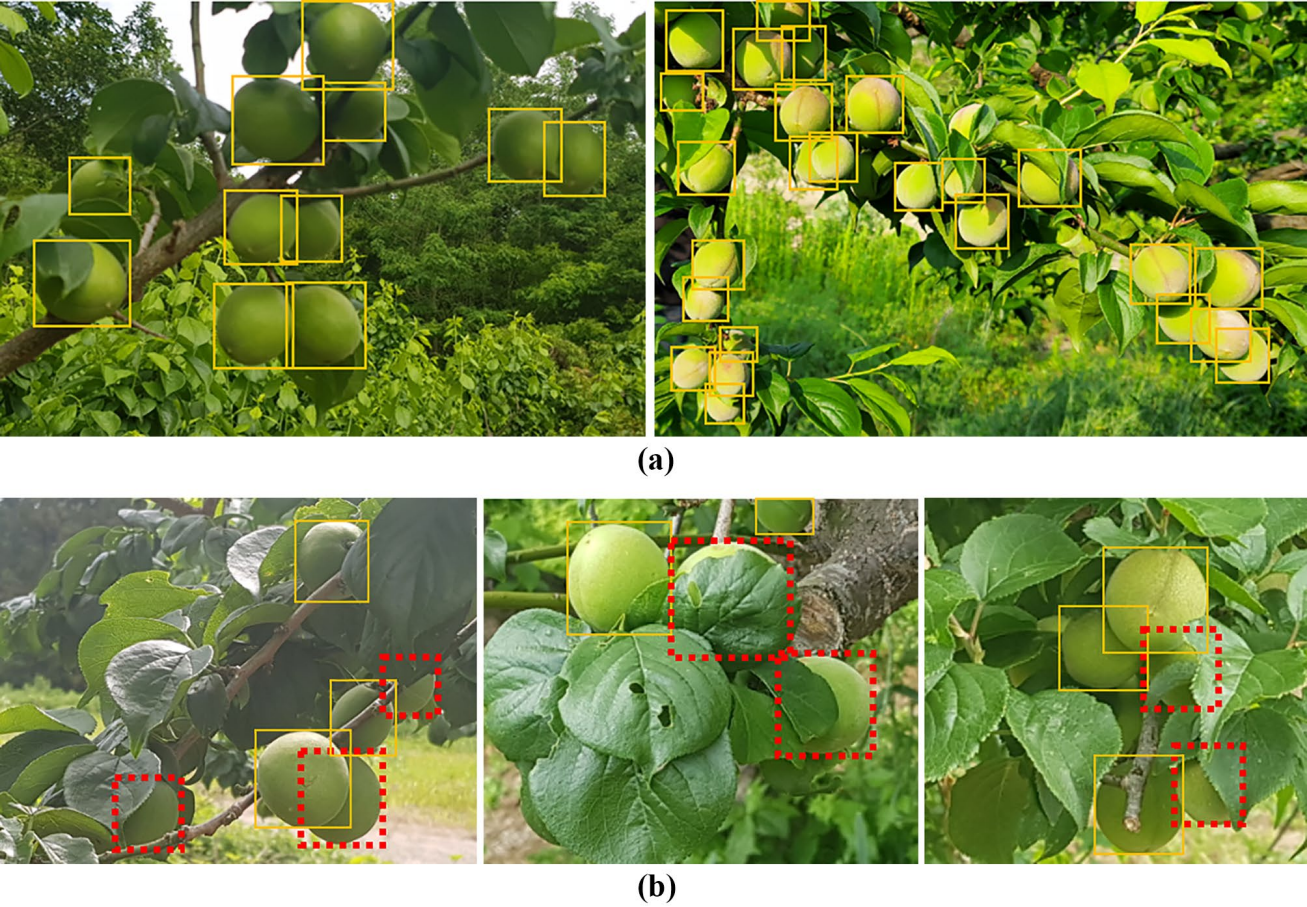
Annotations depending on different conditions: (**a**) Images with small occluded objects with no blurring; (**b**) Images with objects occluded more than 50% (red box, not annotated).

Second, the ground truth boxes of collected *Prunus mume* images were defined by the annotation process for subsequent training of the CNN-based *Prunus mume* detection model. As shown in Fig. 8, the colors of the bushes and leaves, which are similar to those of *Prunus mume* fruits, made identification and annotation difficult. Therefore, in this study, two criteria were established during the annotation process for consistent work. First, annotation was performed only when the image of *the Prunus mume* fruit was not blurred at all, as shown in Fig. 8a. Second, annotation was not performed when more than half of *the Prunus mume* fruit image was occluded by other fruits or leaves, as shown in Fig. 8b. The red dotted line boxes in Fig. 8b show the unannotated *Prunus mume* fruits occluded by branches or leaves. In total, 12,773 *prunus mume* objects were annotated through the annotation process.

### Object detection model training and evaluation

A large volume of training data was required to learn several parameters of deep neural networks. In addition, data augmentation methods have been used to increase the volume and diversity of data by artificially modulating the image for training. In this study, data augmentation methods were applied, including rotating images by 90°, cropping images randomly, adjusting brightness randomly, and flipping images horizontally and vertically. The training models were trained, the validation set for optimizing the detection models, and the test set for evaluating the performance of the model was divided by a ratio, as presented in Table 5.

**Table 5:** Number of images of objects used in training, validation, and testing.

	Train	Validation	Test
Number of Images	431	144	144
Proportion of total images (%)	59.94	20.03	20.03
Number of objects	7940	2391	2442
Proportion of total objects (%)	62.16	18.72	19.12

Deep-learning-based object detection models are composed of a combination of various meta-architectures featuring extractors. Depending on the tasks, the distinct combinations exhibit different accuracies and computing speeds. In this experiment, a total of four models of Faster R-CNN Resnet 101, Efficientdet D4, Retinanet 101, and SSD MobileNet v.2 were trained, and their accuracies and speeds were compared and evaluated. In this study, all networks applied the transfer learning method using pre-trained models with the COCO datasets. We decided that the amount of data was not enough to make our own CNN-based network to learn from scratch model, so we trained the models using the existing pre-trained models for weight initialization. When using the features and weights from pre-trained models, modification of entire layers was applied for more suitable and optimal feature training when detecting *prunus mume* fruits. Python (ver. 3.7, Python Software Foundation, Python Language Reference) was employed as the programming language throughout the entire process, and the training and evaluation part was based on the TensorFlow object detection API. The computing system used for deep neural network training was composed of two GeForce RTX Quadro 6000 (Nvidia Corp., Santa Clara, CA, USA) GPUs and a Xeon Silver 4214R (Intel Corp., Santa Clara, CA, USA) CPU.

Lastly, the performance of each model was evaluated using the test set, and the average precision (AP) index of the object detection model was used for performance evaluation. The average precision was obtained by integrating the precision-recall curve, and precision and recall are defined in Eqs. (1) and (2). True positive (TP) denotes the number of correctly detected *Prunus mume* fruits, and false positive (FP) corresponds to the number of incorrectly detected fruits. False negative (FN) is the number of undetected ground truth objects. The intersection of the union (IOU) threshold between the ground truth box and the detected box determines whether the detected object box is true or false. The higher the IOU threshold, the more accurate the box localization can be recognized as a true detection. In addition, when the IOU threshold increases, the accuracy of the high value with box localization is required for true detection. In this study, the accuracy of the model was evaluated based on the average precision when the IOU threshold was 0.5 and 0.75, respectively.

1 $$Precision = \frac{TP}{{TP + FP}} = \frac{Correctly\, Detected\, Objects}{{All\, Detected\, Objects}}$$

2 $$Recall = { }\frac{TP}{{TP + FN}} = \frac{Correctly\, Detected\, Objects}{{All\, Ground\, Truth\, Objects}}$$

*Prunus mume* fruits are small compared to the entire image size, and generally, object detection models have difficulty detecting small objects. Therefore, the size of the *Prunus mume* fruits in the collected images were divided into three sections (small, medium, and large) to effectively evaluate the detection performance. The analysis of each AP value was based on the IOU threshold of 0.5. The object scale was obtained by dividing the pixel size distribution of the annotated *Prunus mume* fruits into three equal parts. The object scale was classified as follows: objects less than 22 × 22 pixels are small, objects ranging from 22 × 22 to 34 × 34 as medium, and objects larger than 34 × 34 pixels as large.

Furthermore, the image cropping method is commonly used to improve small object detection performance. Therefore, we employed it in this study. As the number of cropping increases, the number of images to be detected also increases, which takes longer. However, the cropping process enlarges the size of the objects compared to the entire image. Therefore, detection accuracy can be improved, particularly for small objects. Therefore, image cropping can be applied to improve performance for tasks that do not require real-time processing. In this study, original images of 1280 × 720 pixels were cropped into 960 × 540, 640 × 360, and 320 × 180 pixels, and models were trained and evaluated for each condition. For instance, a 1280 × 720 pixel-based model indicates a non-cropped model, and four cropped images were obtained for each original image by cropping images based on the four vertices of the original images.

### Growth analysis using depth information

**Figure 9:**
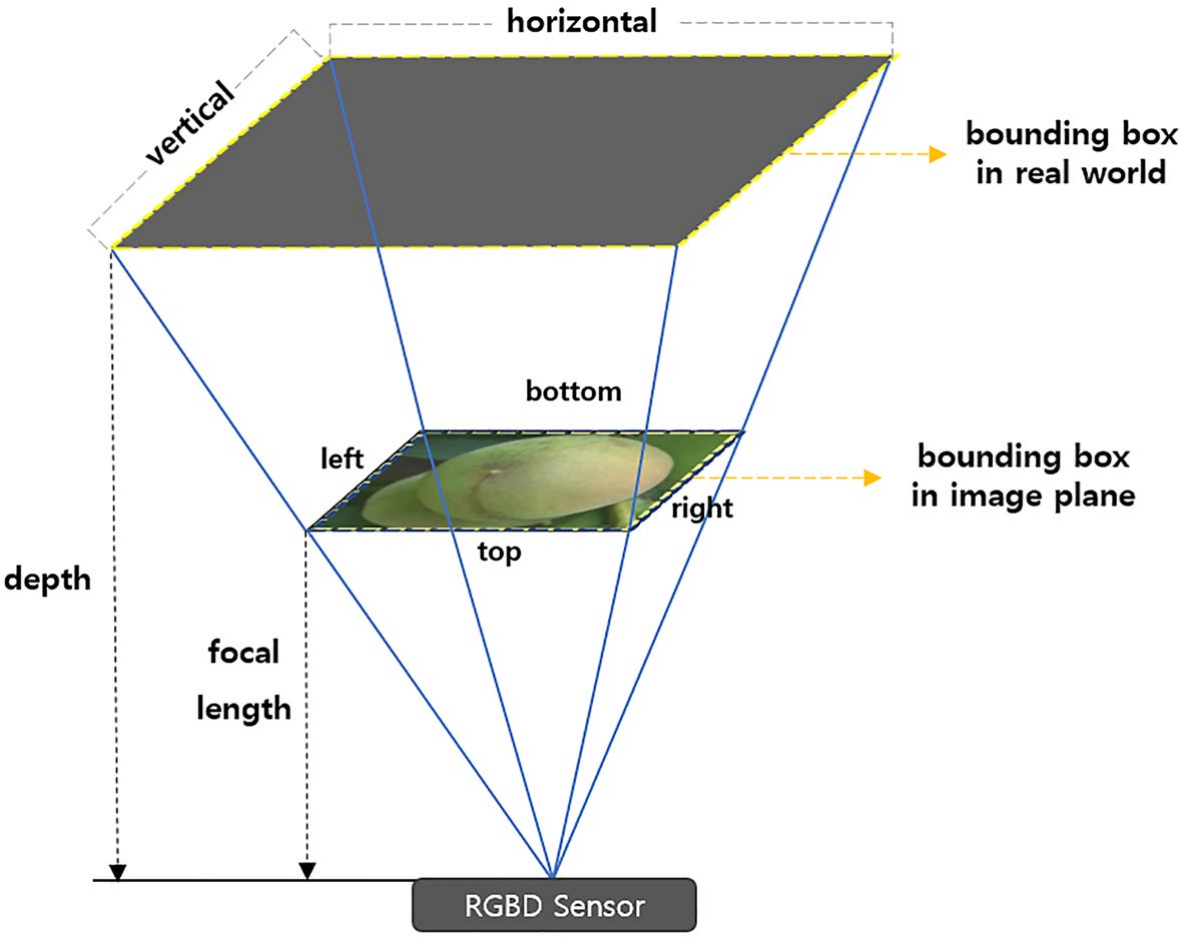
Calculating the diameter of *prunus mume* fruit using trigonometric ratio.

The growth stages of objects were estimated using the relative coordinates of the detected boxes in the RGB image extracted from the CNN-based object detection model. In the process of evaluating the model using the test set, the relative coordinates *(x, y)* and the score values representing the accuracy of each detected box were extracted from the model. Moreover, only the bounding boxes with a score higher than 0.75 were applied to only use high-confidence detections to extract the object’s size more accurately. Subsequently, the vertex coordinates of each detected box were calculated from the center coordinates of each box. Moreover, the coordinates *(x, y)* of each bounding box were matched to the depth image to extract the depth information of the central point, and the detected box information is embedded into the depth map. The diameter of *Prunus mume* fruits was measured using the triangular ratio principle, as shown in Fig. 9, which involves the application of Eqs. (3) and (4) using the depth values of both ends of each detected box and the focal length of the sensor. Since *Prunus mume* fruits have an oval shape, both the long and short axes of each object were calculated, and the verification process was performed based on the long axis. The maximum value of the long axis and the short axis according to Eq. (5) was obtained to represent the diameter of each *Prunus mume* fruit to only extract the long axis information.

3 $$horizontal = \frac{right - left}{{focal}} * depth$$

4 $$vertical = \frac{bottom - top}{{focal}} * depth$$

5 $$long axis = max\left( {vertical, horizontal} \right),\;short axis = min\left( {vertical, horizontal} \right)$$

**Figure 10:**
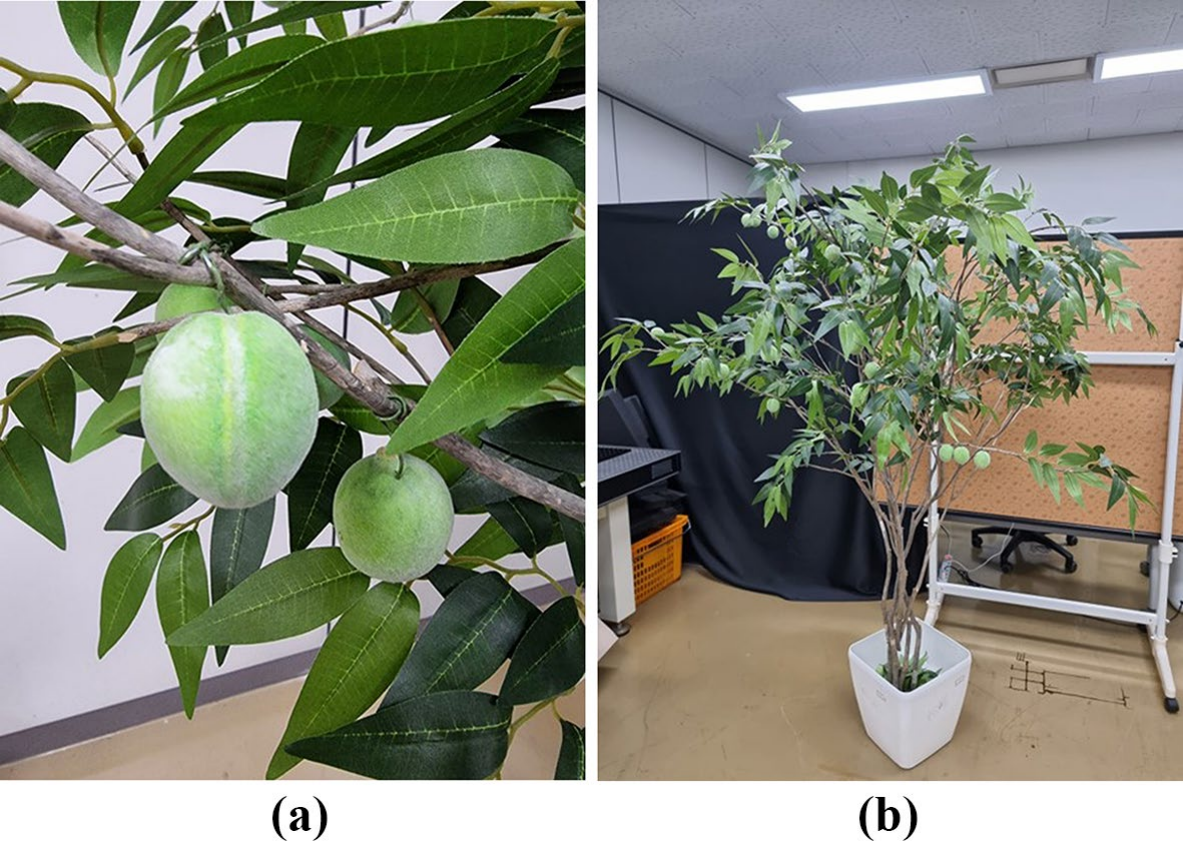
Indoor configuration of artificial *prunus mume*: (**a**) fruits; (**b**) tree.

In this study, a verification process was conducted in both outdoor and indoor environments to verify the developed growth estimation algorithm of *Prunus mume* fruits. For the indoor experiment, as shown in Fig. 10, artificial *Prunus mume* fruits and trees were used. Artificial *Prunus mume* fruits with different diameters were used, as shown in Fig. 10a. The diameter of the artificial *Prunus mume* fruits had various scales ranging from 32 to 37 mm for the long axis and 29 to 34 mm for the short axis. In total, 30 artificial fruits were used for indoor testing, and their average diameter (long axis) was 34 mm. An artificial *Prunus mume* tree for estimating growth stages was constructed, as shown in Fig. 10b, and the artificial *Prunus mume* fruits in Fig. 10a were evenly hung on a 2.1 m high tree. To evaluate the performance of the developed algorithm with an artificially constructed *Prunus mume* tree indoors, data were acquired as similar as possible to the imaging conditions at outdoor *Prunus mume* farms to evaluate the performance of the developed algorithm with an artificially constructed *Prunus mume* tree indoors. For similar imaging conditions, the same RGBD sensor of Intel RealSense D435i was used, and the photographs were captured from a similar distance range where the images were acquired at outdoor farms.

**Figure 11:**
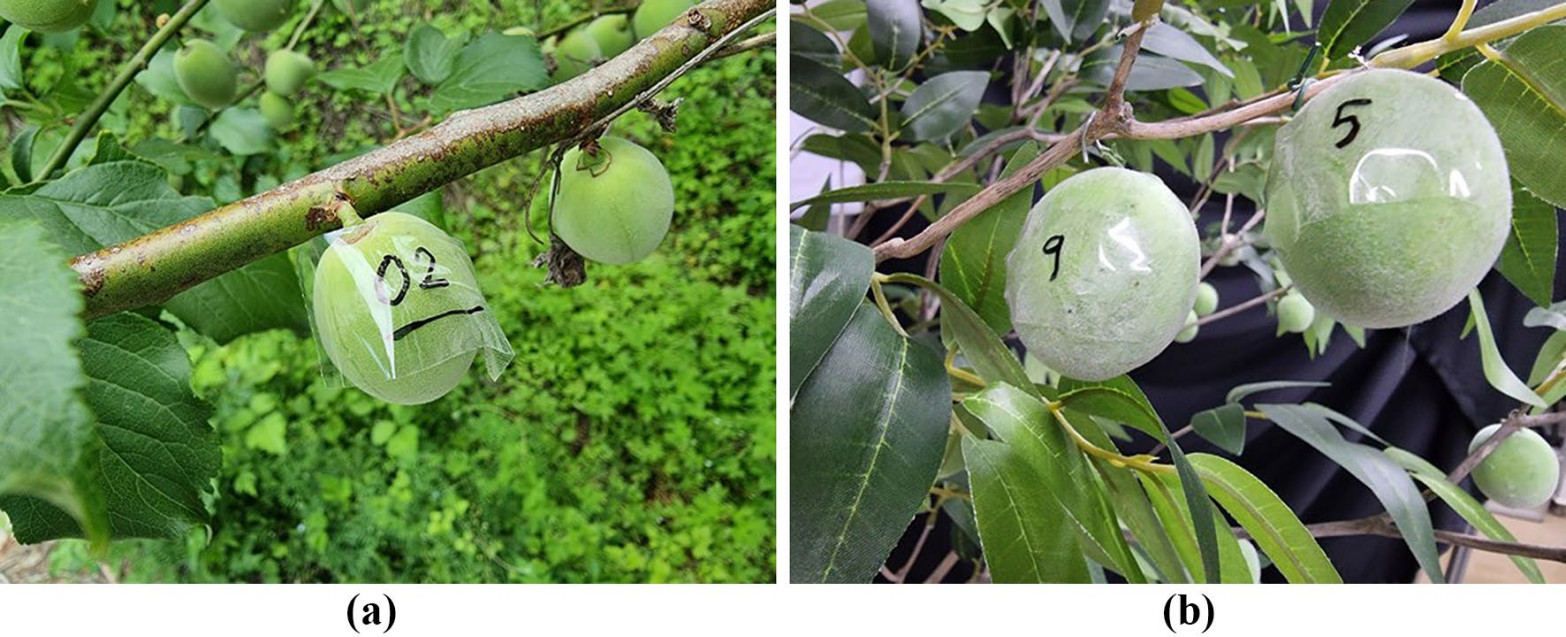
Numbering each *prunus mume* fruit: (**a**) actual outdoor fruits; (**b**) indoor artificial fruits.

**Figure 12:**
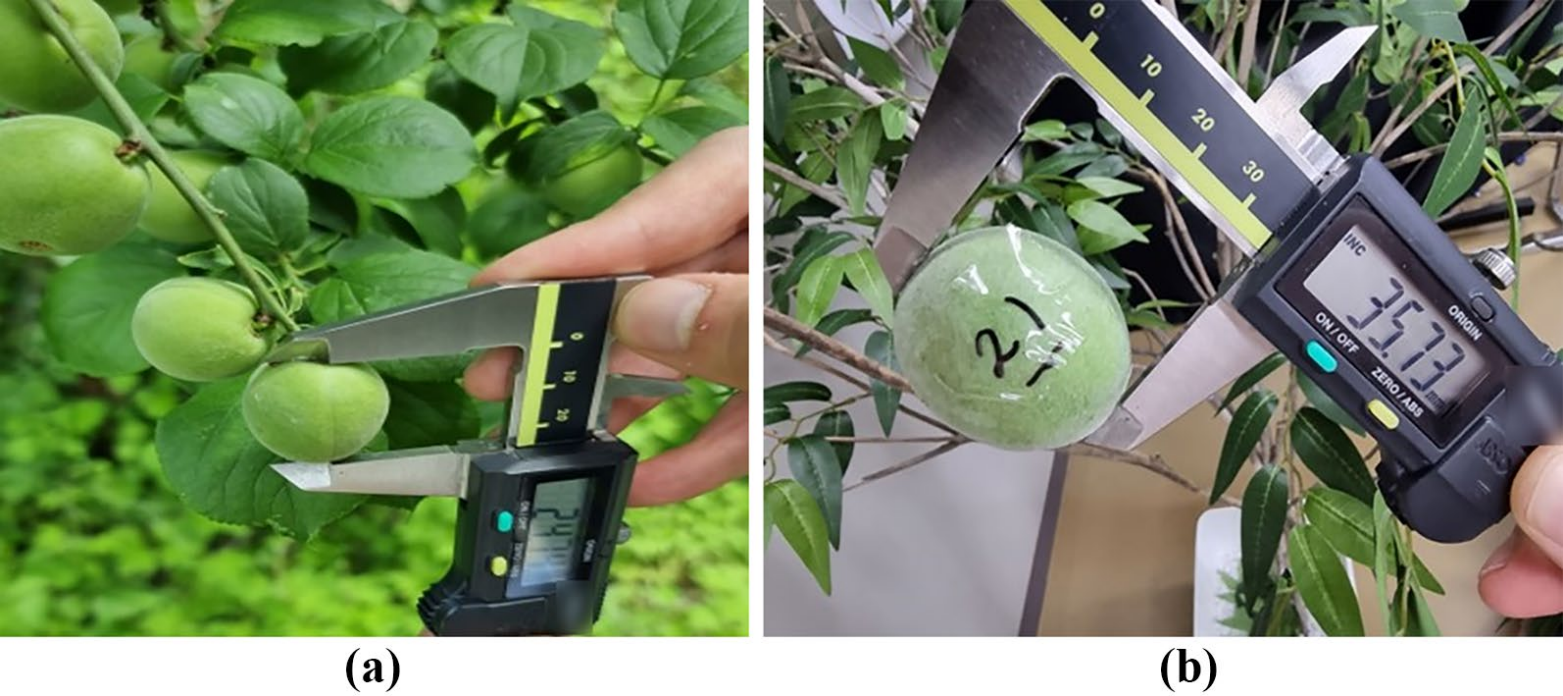
Diameter measurement of *prunus mume* fruits with Vernier Caliper: (**a**) actual outdoor fruits; (**b**) indoor artificial fruits.

**Figure 13:**
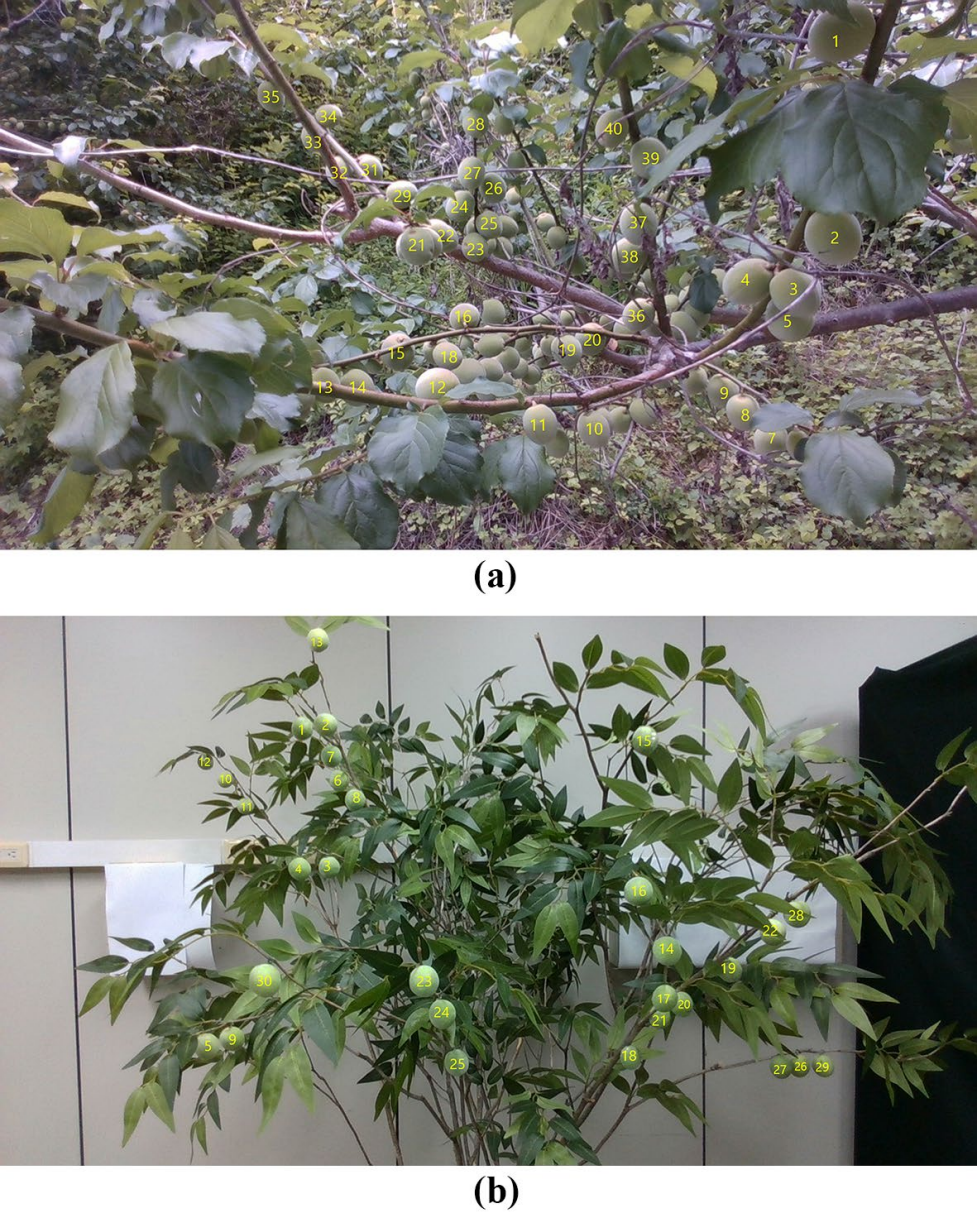
Finding and marking the location of each numbered *Prunus mume* fruit: (**a**) outdoors; (**b**) indoors.

Acquiring images for the verification process in an outdoor environment was initially conducted to build a deep learning model from April to May 2020. Once again, we acquired *Prunus mume* images from the same two local farms (Suncheon City, Republic of Korea) for the verification process in May 2021. Images were collected using the same RGBD camera (Intel RealSense D435i). An additional process of numbering and measuring each *Prunus mume* fruit was performed to verify the growth information measurement. As shown in Fig. 11, each *Prunus mume* fruit in the image was numbered. For each number of fruits, as shown in Fig. 12, the diameter of *Prunus mume* fruits was measured accurately using a Vernier caliper, and these values were recorded for future comparison with calculated diameters using the depth algorithm developed in this study. Subsequently, after determining the location of each numbered fruit on the image, we marked the numbers on each *Prunus mume* fruit on the acquired image, as shown in Fig. 13. Eventually, five *prunus mume* images, four outdoor images, and one indoor image were used as the verification process.

## Data Availability

The data that support the findings of this study are available from Ghiseok Kim, but restrictions apply to the availability of these data, which were used under license for the current study, and so are not publicly available. Data are however available from the authors upon reasonable request and with permission of Ghiseok Kim.

## References

[1] Moghadam, P. *et al.* Plant disease detection using hyperspectral imaging. In *DICTA 2017 - 2017 International Conference on Digital Image Computing: Techniques and Applications* vols. 2017-December 1–8 (Institute of Electrical and Electronics Engineers Inc., 2017).

[2] Chetan Dwarkani, M., Ganesh Ram, R., Jagannathan, S., Priyatharshini, R. Smart farming system using sensors for agricultural task automation. In *Proceedings - 2015 IEEE International Conference on Technological Innovations in ICT for Agriculture and Rural Development, TIAR 2015* 49–53 (Institute of Electrical and Electronics Engineers Inc., 2015). Doi: 10.1109/TIAR.2015.7358530.

[3] Migdall, S., Klug, P., Denis, A., Bach, H. The additional value of hyperspectral data for smart farming. In *International Geoscience and Remote Sensing Symposium (IGARSS)* 7329–7332 (2012). Doi:10.1109/IGARSS.2012.6351937.

[4] Meyer MH (2016). Importance of horticulture and perception as a career. J. Am. Soc. Horticu. Sci..

[5] Grift T, Zhang Q, Kondo N, Ting KC (2008). A review of automation and robotics for the bio-industry. J. Biomech. Eng..

[6] Posadas B (2012). Economic impacts of mechanization or automation on horticulture production firms sales, employment, and workers’ earnings, safety, and retention. HortTechnology.

[7] Chaerle L, van der Straeten D (2000). Imaging techniques and the early detection of plant stress. Trends Plant Sci..

[8] Liang L (2015). Estimation of crop LAI using hyperspectral vegetation indices and a hybrid inversion method. Remote Sens. Environ..

[9] Kim S-H (2018). Estimation of moisture content in cucumber and watermelon seedlings using hyperspectral imagery. Prot. Hortic. Plant Factory.

[10] Basak JK, Qasim W, Okyere FG, Khan F, Lee YJ, Kim HT (2019). Regression analysis to estimate morphology parameters of pepper plant in a controlled greenhouse system. J. Biosyst. Eng..

[11] Birwal P, Deshmukh G, Saurabh SP (2017). Nutrition and population health citation. Rev. Art. J. Food.

[12] Igwe EO, Charlton KE (2016). A Systematic review on the health effects of plums (*Prunus domestica* and *Prunus salicina*). Phytother. Res..

[13] Arjmandi BH (2017). Bone-protective effects of dried plum in postmenopausal women: Efficacy and possible mechanisms. Nutrients.

[14] Kim HR, Kim ID, Dhungana SK, Kim MO, Shin DH (2014). Comparative assessment of physicochemical properties of unripe peach (Prunus persica) and Japanese apricot (Prunus mume). Asian Pac. J. Trop. Biomed..

[15] Wang Y, Chen X, Zhang Y, Chen X (2012). Antioxidant activities and major anthocyanins of myrobalan plum (Prunus cerasifera Ehrh.). J Food Sci.

[16] Vizzotto M, Cisneros-Zevallos L, Byrne DH, Ramming DW, Okie WR (2007). Large variation found in the phytochemical and antioxidant activity of peach and plum germplasm. J. Am. Soc. Hortic. Sci..

[17] Jung B-G (2010). Immune-enhancing effect of fermented maesil (Prunus mume Siebold & Zucc.) with probiotics against bordetella bronchiseptica in mice. J. Vet. Med. Sci..

[18] Adachi M (2007). The “Prunus mume Sieb et. Zucc” (Ume) is a rich natural source of novel anti-cancer substance. Int. J. Food Prop..

[19] Go MR, Kim HJ, Yu J, Choi SJ (2018). Toxicity and toxicokinetics of amygdalin in maesil (Prunus mume) syrup: Protective effect of maesil against amygdalin toxicity. J. Agric. Food Chem..

[20] Milošević T, Milošević N, Glišić I (2013). Agronomic properties and nutritional status of plum trees (Prunus domestica L.) influenced by different cultivars. J. Soil Sci. Plant Nutr..

[21] Ahmad J, Jan B, Farman H, Ahmad W, Ullah A (2020). Disease detection in plum using convolutional neural network under true field conditions. Sensors.

[22] Choi DS, Ko SJ, Ma GC, Kim HJ, Kim DE, Kim HW (2015). Damage, occurrence, and optimal control period of Eurytoma maslovskii affecting Japanese Apricot (Prunus mume) fruits in Jeonnam province. Korean J. Appl. Entomol..

[23] Lee JD, Kim GS, Kong HJ, Cho SY, Kim HJ (2016). Development of growth and cultivation environment monitoring system to determine the right time to control plums. J. Korean Soc. Agric. Mach..

[24] Tian Y (2019). Apple detection during different growth stages in orchards using the improved YOLO-V3 model. Comput. Electron. Agric..

[25] Putra BTW, Amirudin R, Marhaenanto B (2022). The evaluation of deep learning using convolutional neural network (CNN) approach for identifying Arabica and Robusta coffee plants. J. Biosyst. Eng..

[26] Krizhevsky A, Sutskever I, Hinton GE (2017). ImageNet classification with deep convolutional neural networks. Commun. ACM.

[27] Rasti S (2020). Crop growth stage estimation prior to canopy closure using deep learning algorithms. Neural Comput. Appl..

[28] Teimouri N (2018). Weed growth stage estimator using deep convolutional neural networks. Sensors.

[29] Stokes JM (2020). A deep learning approach to antibiotic discovery. Cell.

[30] Rahhal MMA (2016). Deep learning approach for active classification of electrocardiogram signals. Inf. Sci..

[31] Paulo Marques do Nascimento, P. Applications of deep learning techniques on NILM. *Diss. Universidade Federal do Rio de* Janeiro (2016).

[32] Ammour N (2017). Deep learning approach for car detection in UAV imagery. Remote Sens..

[33] Chen C-H, Kung H-Y, Hwang F-J (2019). Deep learning techniques for agronomy applications. Agronomy.

[34] Hong SJ, Han Y, Kim SY, Lee AY, Kim G (2019). Application of deep-learning methods to bird detection using unmanned aerial vehicle imagery. Sensors.

[35] Simonyan, K. & Zisserman, A. Very deep convolutional networks for large-scale image recognition. In *3rd International Conference on Learning Representations, ICLR 2015 - Conference Track Proceedings* (International Conference on Learning Representations, ICLR, 2015).

[36] Joo Y-D (2017). Drone image classification based on convolutional neural networks. J. Inst. Internet, Broadcast. Commun..

[37] Bharati, P., Pramanik, A. Deep learning techniques—R-CNN to Mask R-CNN: A survey. Advances in Intelligent Systems and Computing **999**, 657–668 (Springer, 2020)

[38] Girshick, R. Fast R-CNN. In *Proceedings of the IEEE Conference on Computer Vision (ICCV),* 1440–1448 (2015).

[39] Ren S, He K, Girshick R, Sun J (2017). Faster R-CNN: Towards real-time object detection with region proposal networks. IEEE Trans. Pattern Anal. Mach. Intell..

[40] He, K., Gkioxari, G., Dollár, P. & Girshick, R. Mask R-CNN. In *Proceedings of the IEEE International Conference on Computer Vision (ICCV),* 2961–2969 (2017).

[41] Lee HS, Shin BS (2020). Potato detection and segmentation based on mask R-CNN. J. Biosyst. Eng..

[42] Liu, W. et al. SSD: Single shot multibox detector. Lecture Notes in Computer Science (including subseries Lecture Notes in Artificial Intelligence and Lecture Notes in Bioinformatics) vol. 9905 LNCS 21–37 (Springer Verlag, 2016)

[43] Lin, T.-Y., Goyal, P., Girshick, R., He, K. & Dollár, P. Focal loss for dense object detection. In *Proceedings of the IEEE International Conference on Computer Vision*, 2980–2988 (2017).

[44] Tan, M., Pang, R. & Le, Q. v. EfficientDet: Scalable and efficient object detection. In *Proceedings of the IEEE/CVF Conference on Computer Vision and Pattern Recognition (CVPR),* 10781–10790 (2020).

[45] Jang E-C (2021). 3D image processing for recognition and size estimation of the fruit of plum (Japanese apricot). J. Korea Contents Assoc..

